# Incorporation of 1-methylcyclopropene and salicylic acid improves quality and shelf life of winter jujube (*Zizyphus jujuba* Mill. cv. Dongzao) through regulating reactive oxygen species metabolism

**DOI:** 10.3389/fnut.2022.940494

**Published:** 2022-07-25

**Authors:** Weida Zhang, Jiawei Kang, Wanting Yang, Huijing Guo, Minrui Guo, Guogang Chen

**Affiliations:** ^1^College of Food Science and Technology, Shihezi University, Shihezi, China; ^2^Institute of Agricultural Products Processing, Xinjiang Academy of Agricultural Reclamation Sciences, Shihezi, China

**Keywords:** 1-MCP, winter jujube, shelf quality, enzyme activity, SA

## Abstract

Winter jujube fruit is susceptible to aging, peel reddening, dehydration, shrinkage, and tissue softening during shelf life after it is removed from the cold storage conditions. In this study, the effects of 1-methylcyclopropene (1-MCP) and salicylic acid (SA) on the quality of winter jujube fruit during shelf life were investigated by measuring physiological indexes and the activities of antioxidant enzymes and enzymes related to reactive oxygen species (ROS) metabolism of winter jujube fruit. The results showed that 1-MCP treatment and SA treatment suppressed weight loss, respiratory rate, malondialdehyde (MDA) content, H_2_O_2_ content, and ${\text{O}}_{2}^{-\cdot }$ production rate, but improved firmness, color difference (Δ*E*), soluble solid content (SSC), titratable acidity (TA), superoxide dismutase (SOD), peroxidase (POD), catalase (CAT), ascorbate peroxidase (APX), glutathione reductase (GR), phenylalanine ammonia-lyase (PAL) activities, ascorbic acid content, glutathione content, total phenolic content, and total flavonoid content in comparison with the control. Particularly, the combined treatment of 1-MCP and SA (1-MCP+SA treatment) showed the maximum efficacy compared to the 1-MCP treatment and SA treatment alone. 1-MCP+SA treatment exhibited the best preservation effect, followed by SA treatment and 1-MCP treatment. Thus, the combined treatment of 1-MCP and SA is an effective approach to maintain the postharvest quality of winter jujube fruit and extend the shelf life.

## Introduction

Winter jujube (*Ziziphus jujuba* Mill. cv. Dongzao) is one of the important fruits in Xinjiang, China, and is popular for its flavor and texture. It is rich in biologically active substances, such as flavonoids and polyphenols, which have disease-preventing and health-promoting functions (1, 2). Due to the short shelf life at room temperature, winter jujube fruits are commonly stored at low-temperature conditions to improve the commercial quality of fruit. However, once out of the low-temperature storage, fruit aging, peel reddening, dehydration, shrinkage, and tissue softening are observed, which seriously affect the sensory quality and nutritional quality, and reduce the commercial value and shelf life (3). So, simple low-temperature storage is not sufficient for the preservation of winter jujube fruit. At present, some preservation techniques have also been reported for the postharvest shelf life extension of winter jujube fruit, including reduced pressure storage, https://www.sciencedirect.com/topics/food-science/modified-atmosphere-packaging controlled atmosphere storage, and chemical preservation (1).

Chemical preservatives have been widely used in fruit storage and preservation. The application of chemical preservatives can delay fruit senescence, prevent rot, kill pathogenic bacteria, reduce respiration rate, and slow down water evaporation (3). Due to greater public awareness for health, the environment and international regulations have accentuated the usage of generally recognized as safe (GRAS) compounds. SA and 1-MCP have been classified by the U.S. Food and Drug Administration (FDA) as GRAS compounds and are considered safe for humans (4). SA is a small molecular phenolic acid in plants. As an endogenous signal molecule and a hormone, it participates in the regulation of plant physiological, biochemical, and metabolic processes, and could improve fruit resistance to pathogens (5). A large number of studies have shown that SA could maintain the postharvest quality of pomegranate (6), lemon (7), citrus (8), apricot (9), and peach (10). Zhang et al. (5) found that SA treatment could maintain the firmness and sensory quality of wolfberry fruit, and delay fruit ripening and senescence. Baswal et al. (4) found that SA and 1-MCP treatment could improve firmness, inhibit weight loss, and prolong the cold storage life in citrus fruit. As another widely used chemical preservative, 1-MCP can superiorly suppress ethylene action and respiratory climacteric in fruits by competitively binding to ethylene receptors, thus delaying fruit senescence and prolonging shelf life (11). Studies have shown that 1-MCP is a non-toxic, odorless, and stable substance that could better preserve postharvest fruit quality. It has been applied to the storage and preservation of apple (12), banana (13), durian (14), melon (15), kiwifruit (16), sweet cherry (17), etc.

Many studies have confirmed that 1-MCP and SA have good preservation effects on fruits. However, to date, there is no report on the effects of the combined treatment of 1-MCP and SA on winter jujube fruit during shelf life. Therefore, the effect of the combined treatment of 1-MCP and SA on the storage quality and preservation effect of winter jujube fruit was evaluated in this study by analyzing the physicochemical indexes and related enzyme activities during shelf life. Our research will provide technical references for prolonging shelf life and improving the commercial quality of winter jujube fruit.

## Materials and Methods

### Fruit materials and treatment

Winter jujube (*Zizyphus jujuba* Mill. cv. Dongzao) fruits (white ripe stage, soluble solid content 23.5%) were harvested from an orchard of Korla in Xinjiang, China, and immediately transported to the laboratory located in Shihezi University. Winter jujube fruits were pre-cooled at 5°C for 24 h. Then fruits with no pests and diseases, no mechanical damage, and uniform color were selected and stored at 4 ± 1 °C at relative humidity (RH) of 85–95% for 1 month. Shelf life began after the cold storage. All winter jujube fruits were randomly divided into four groups and then treated according to the following experimental design.

**Figure 1:**
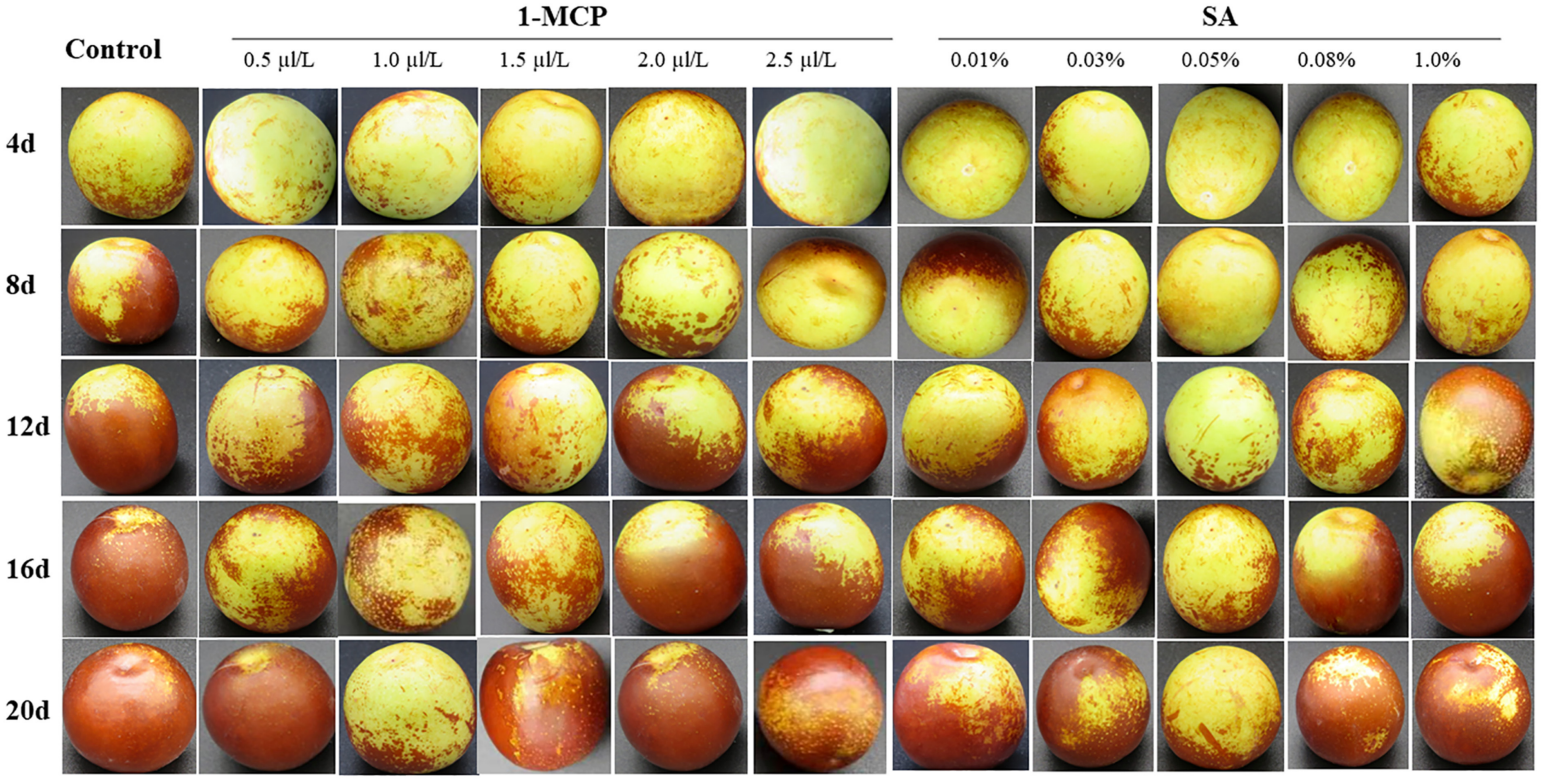
Changes in cuticle color in winter jujube fruit treated with different concentrations of 1-methylcyclopropene (1-MCP) (0–2.5 μl/L) and salicylic acid (SA) (0–1.0%) during shelf life at 4 ± 1 °C for 20 d.

The chemicals 1-MCP and SA were purchased from Beihua Chemical Reagent Co., Ltd., Beijing, China. In preliminary experiment, different concentrations of 1-MCP solution (0.5, 1.0, 1.5, 2.0, and 2.5 μL L^−1^) were prepared. Then, winter jujube fruits were fumigated for 24 h at 20 ± 2°C and 85–95% RH. Fifteen winter jujube fruits were treated for each concentration and each duration. Similarly, different concentrations of SA solution (0.01%, 0.03%, 0.05%, 0.08%, and 1.0%) were prepared. Then, winter jujube fruits were soaked for 5 min at 20 ± 2°C and 85–95% RH. Fifteen winter jujube fruits were treated for each concentration and each duration. According to the results, considering the preservation effect, 1.0 μl L^−1^ concentration of 1-MCP was selected for the 1-MCP treatment with a fumigating time of 24 h, while 0.05% concentration of SA was selected for the SA treatment with a soaking time of 5 min (Figure 1).

Four groups of winter jujube fruit were tagged: (1) Control group (fruits were untreated), (2) 1-MCP group (fruits were fumigated with 1.0 μl L^−1^ of 1-MCP solution for 24 h), (3) SA group (fruits were soaked in 0.05% SA solution for 5 min), and (4) 1-MCP + SA group (fruits were soaked in 0.05% SA solution for 5 min and then fumigated with 1.0 μl L^−1^ of 1-MCP solution for 24 h). Each group had 60 fruits, and 20 fruits in each group served as a replicate. Subsequently, all the winter jujube fruits were stored in refrigerated display cabinets (4 ± 1°C and 85–95% RH) to simulate the shelf-life storage. Three fruits (replicates) were sampled from each group on days 0, 4, 8, 12, 16, and 20 during shelf life for analysis, and the mean value was calculated.

### Measurement of physicochemical parameters

The surface color of fruits was measured by using a chroma meter YS3060 (Sanenshi Scientific Instruments Co., Ltd., Shenzhen, China). Fifteen winter jujube fruits were randomly selected from each group to measure the surface color at the fruit equator. Each fruit was measured three times, and the average value was calculated. The surface color of winter jujube fruit in the control group was used as the blank (Δ*E*=*0*), and the values of Δ*E* of all groups were recorded.

The respiration rate was detected by measuring CO_2_ production (15). Winter jujube fruits (~500 g) were transferred into a glass jar (1 L) and enclosed for 15 min prior to CO_2_ sampling, followed by the detection with an infrared gas analyzer (FS-3080A, Shijiazhuang Fansheng Scientific Instruments Co., Ltd., Hebei, China). The results of respiration rate were expressed as ng kg^−1^ s^−1^. Measurement was conducted for three times.

The weight loss of winter jujube fruit was determined using an electronic balance. The calculation formula was as follows:


$$\begin{array}{l} \text{Weight loss }\left(\%\right)=\frac{m_{1}-m_{2}}{m_{1}}\times 100\% \end{array}$$

where *m*_1_ is the original weight (g) of winter jujube, and *m*_2_ is the weight (g) of winter jujube on the sampling date.

Fruit firmness was determined with a GY-4 sclerometer (Zhejiang Tuopuyunnong Scientific Instruments Co., Ltd., Hangzhou, China). Winter jujube was peeled, and the maximum force (N) was measured. Five fruits were measured at each sampling date, and the firmness of each fruit was determined at the fruit equator for three times. Then, the average value was calculated.

The SSC of winter jujube fruit was determined with the GMK-701R digital refractometer (G-Won Hitech, Seoul, Korea). Five seedless winter jujube fruits were ground into pulp in a mortar, and the filtrate was taken after filtration for the determination of SSC. The results were expressed as a percentage (%). Measurement was conducted for three times. TA was determined using the same filtrate with a pH meter by titrating with 0.1 M NaOH to obtain a pH of 8.2, and the results were expressed as a percentage (%).

Ethylene production was measured according to the method of Nguyen et al. (15) with some modifications. Fifteen fruits were enclosed in a 1 L airtight jar for 1 h, and then Shimadzu gas chromatography with flame ionization detector (FID) and GDX-502 packed column was used to analyze the sample. Results were expressed as C_2_H_4_ μL·kg^−1^·s^−1^. Measurement was conducted for three times.

### Measurement of MDA content, H_2_O_2_ content, and O_2_^−·^ generation rate

Winter jujube fruit samples (50 g) were ground into powder using a pre-cooled grinder. Pre-cooled winter jujube samples (1 g) were used for measuring MDA content, H_2_O_2_ content, and ${\text{O}}_{2}^{-\cdot }$ generation rate following the method of Tang et al. (18), and the final results were expressed as μmol^−1^ kg, μmol^−1^ kg, and μmol^−1^ kg ^−1^ s, respectively, based on the fresh weight.

### Measurement of enzymes

The activities of SOD and CAT were determined using pre-cooled winter jujube samples (1 g) according to the method described by Tang et al. (18). One unit (U) of SOD activity was defined as the amount of enzyme that causes 50% inhibition of nitroblue tetrazolium (NBT), and one U of CAT activity was defined as the degradation of 1 μmol of H_2_O_2_ per kg of winter jujube tissue per minute at 240 nm. The results were expressed as U·kg^−1^ based on the fresh weight.

The activities of POD and APX were assayed according to Chu et al. (19), and the results were expressed as U·kg^−1^ on the basis of fresh weight. One U of POD and APX activities was defined as an increase in absorbance by 0.01 per minute at 470 and 290 nm, respectively.

The GR activity was measured according to the method of Smith et al. (20), and the results were expressed as U·kg^−1^ on the basis of fresh weight. One U of GR activity was defined as the oxidation of 1 nmol of NADPH per minute.

The PAL activity was measured based on the method of Huang et al. (21), and the results were expressed as U·kg^−1^ on the basis of fresh weight. An increase in absorbance of 0.01 per minute at 290 nm was considered as one U of PAL activity.

### Measurement of non-enzymatic antioxidant systems

The ascorbic acid, total phenolic, and total flavonoid contents were measured according to the method of Chu et al. (19), and the results were expressed as g kg^−1^ on a fresh weight basis. The glutathione content was measured according to the method of Tang et al. (18), and the result was expressed as g kg^−1^ on a fresh weight basis.

### Statistical analysis

The data were processed by one-way analysis of variance (ANOVA) using SPSS software (version 24.0, IBM Corp., Armong, NY, USA). The results are presented as mean ± standard deviation (*n* = 3). The comparison of means was performed using Duncan's multiple range test (DMRT). Results were considered statistically significant at *P* < 0.05. Correlations among indicators were determined using Pearson's correlation test.

## Results and discussion

### 1-MCP+SA treatment changed the surface color of winter jujube fruit during shelf life

According to the screening results of the pre-experiment (Figure 1), 1.0 μl L^−1^ 1-MCP treatment and 0.05% SA treatment can effectively inhibit the reddening of winter jujube fruits. Considering the preservation effect, 1.0 μl L^−1^ 1-MCP and 0.05% SA concentrations were selected for the follow-up experiments.

**Figure 2:**
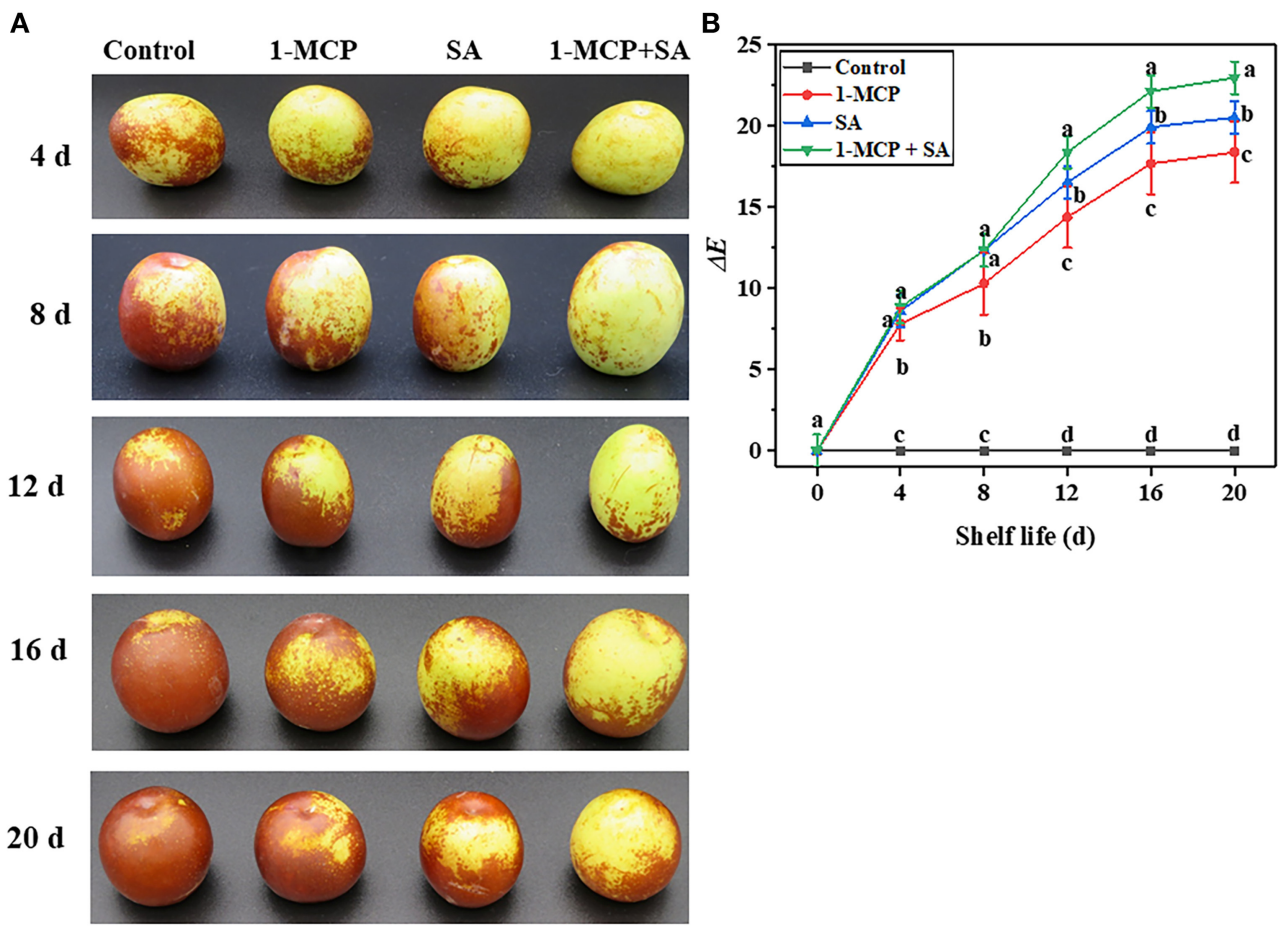
Effects of 1-methylcyclopropene (1-MCP), salicylic acid (SA), and 1-MCP and SA combined treatments on fruit appearance **(A)** and color value Δ*E* **(B)** of winter jujube during shelf life at 4 ± 1°C for 20 d. The data presented are the mean values of three replicates; Vertical bars represent the standard errors of mean values; values followed by different superscripts (a–d) are significantly different (*P* < 0.05) on the same sampling date.

Surface color is one of the important indexes to evaluate fruit sensory quality (15). In this experiment, with the extension of shelf life, the surface color of winter jujube fruit changed from green to red (Figure 2A). The fruits showed different degrees of reddening in the four groups (control group >1-MCP group > SA group > 1-MCP+SA group). This indicates that the combined treatment of 1-MCP and SA can effectively slow down color change and maintain fruit sensory quality. Besides, it was found that with the extension of shelf life, the Δ*E* value of winter jujube fruit of the 1-MCP, SA, and 1-MCP+SA groups showed a trend toward continuous rise and reached the maximum value on day 16 (17.69, 19.93, and 22.15, respectively) (Figure 2B). This may be due to the fact that 1-MCP and SA could inhibit the degradation of chlorophyll and the accumulation of carotenoids, and can further delay fruit reddening and ripening to varying degrees, thus leading to different Δ*E* values (15). Among them, the 1-MCP+SA group had the lowest degree of fruit redness, so it had the largest Δ*E* value. After shelving for 16 days, the fruits in the control group were almost completely red (Figure 2A), while those in the 1-MCP+SA, SA, and 1-MCP groups continued to turn red. Therefore, the Δ*E* value of 1-MCP+SA, SA, and 1-MCP groups became larger in the later period of shelf life. These results indicate that the combined treatment of 1-MCP and SA could more obviously slow down the color change of winter jujube fruit than the 1-MCP treatment and SA treatment alone.

### 1-MCP+SA treatment inhibited the increase of respiration rate of winter jujube fruit during shelf life

**Figure 3:**
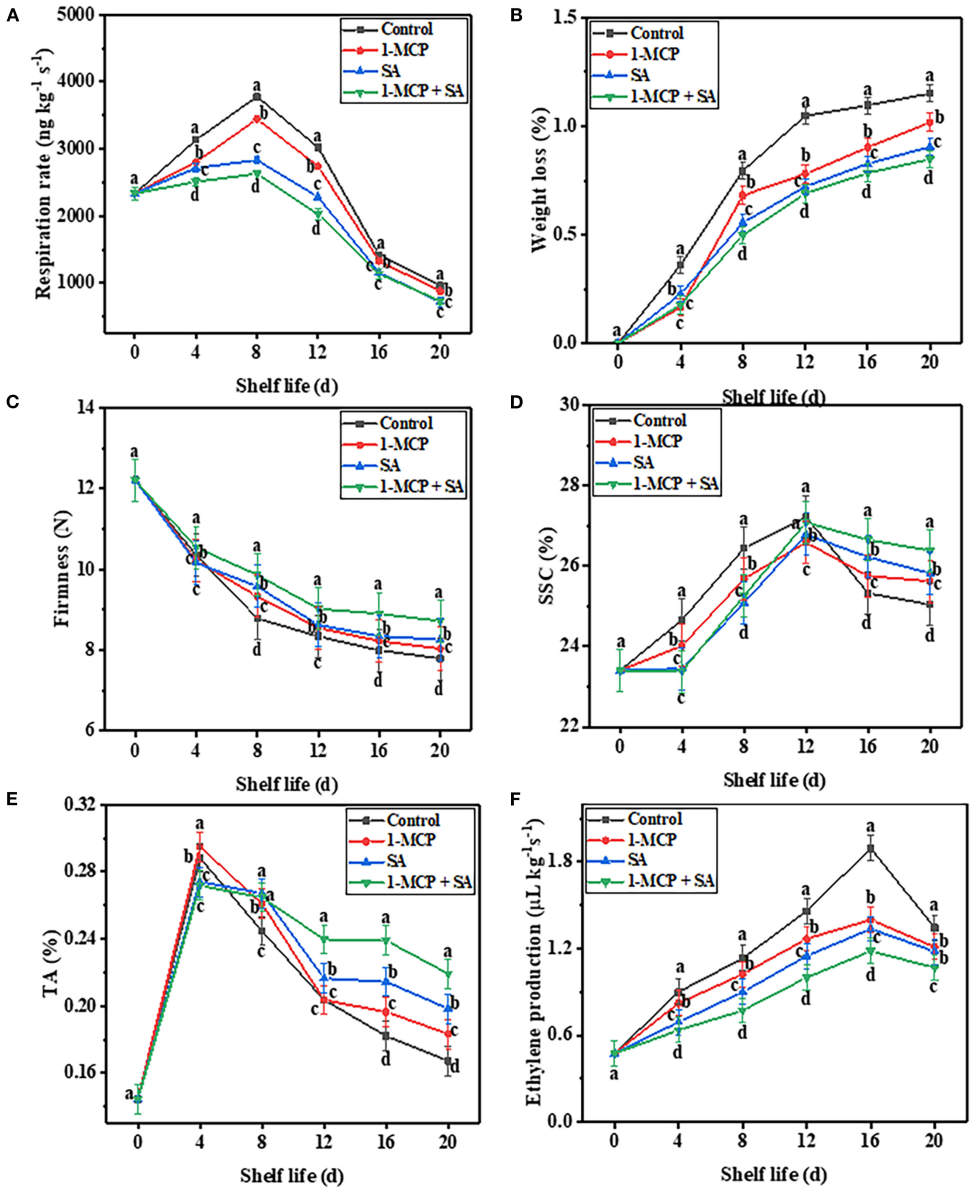
Effects of 1-methylcyclopropene (1-MCP), salicylic acid (SA), and 1-MCP and SA combined treatments on respiratory rate **(A)**, weight loss **(B)**, firmness **(C)**, SSC **(D)**, TA **(E)**, and ethylene production **(F)** of winter jujube fruit during shelf life at 4 ± 1°C for 20 d. SSC, soluble solid content; TA, titratable acidity. The data presented are the mean values of three replicates; vertical bars represent the standard errors of the mean values; values followed by different superscripts (a-d) are significantly different (*P* < 0.05) on the same sampling date.

Respiration is the most important metabolic activity that consumes SSC and TA in postharvest fruit (3). It has been used as a crucial index to explore the changes in physiology and storage quality of postharvest fruit. As shown in Figure 3A, the respiratory rate of fruit in the four groups showed a trend toward rapid increase from day 0 to day 8, and reached the peak (3,780.58 (control), 3,444.47 (1-MCP), 2,833.36 (SA), and 2,630.58 (1-MCP+SA) ng kg^−1^ s^−1^) on day 8. Then the respiration rate of fruit began to decrease after day 8, and decreased to 963.90 (control), 877.78 (1-MCP), 716.67 (SA), and 730.56 (1-MCP+SA) ng kg^−1^ s^−1^ on day 20. During the whole shelf life, the respiration rate of the fruit was always higher in the control group than in the other groups. These results indicate that the 1-MCP, SA, and 1-MCP+SA treatments could effectively inhibit the increase in the respiratory rate of winter jujube fruit, and the 1-MCP+SA treatment exhibited the best effect. In addition, it was noticed that SA treatment showed a better inhibition effect on the respiration rate than the 1-MCP treatment. This may be due to the fact that the 1-MCP treatment had no effects on CO_2_, while SA treatment could inhibit CO_2_ production pathways, which further slows down the respiration rate (22). Similar results have also been found in goji (5), apricot (9), and cherry (23). The excessive accumulation of CO_2_ and low concentration of O_2_ in the 1-MCP treatment group may also contribute to a small reverse inhibition of fruit respiration metabolism during storage, which also supports our results (24). Therefore, SA has a better effect on delaying the maturation and senescence of winter jujube fruit than 1-MCP.

### 1-MCP+SA treatment changed the weight loss and firmness of winter jujube during shelf life

During the shelf life, winter jujube fruit continuously lost water, shrank, and became smaller due to respiration and transpiration. As shown in Figure 3B, with the extension of shelf life, the weight loss of winter jujube fruit increased slowly in the four groups. The weight loss of the control group reached the maximum value (1.15%) on day 20. 1-MCP, SA, and 1-MCP+SA treatments inhibited the weight loss of winter jujube compared with the control group. On day 20, the weight loss in 1-MCP, SA, and 1-MCP+SA groups decreased by 11.3, 20.9, and 26.1%, respectively, compared with that in the control group (*P* < 0.05). Therefore, the inhibition effect of 1-MCP+SA treatment on fruit weight loss is better than that of 1-MCP and SA treatments alone. This may be due to the synergistic effect of 1-MCP and SA. Besides, the inhibition effect of the 1-MCP treatment was poorer than that exhibited by the SA treatment. This may be due to the fact that the response to 1-MCP is dependent on the cultivar type, concentration, and storage conditions (4).

Fruit firmness is an important parameter for evaluating fruit ripening, senescence, and sensory quality during storage and shelf life. Figure 3C shows the variation trend of fruit firmness of winter jujube. In this study, the firmness of fruit in the control group decreased with the extension of shelf life and reached the lowest value (7.79 N) on day 20. Both SA and 1-MCP treatments could maintain fruit firmness to varying degrees, and SA treatment had a better effect than 1-MCP treatment. The 1-MCP+SA treatment showed the most significant effect on maintaining fruit firmness (*P* < 0.05), with the firmness on day 20 accounting for 71.4% (8.72 N) of the initial value. The softening degree of fruit in SA, 1-MCP, and 1-MCP+SA groups were significantly lower than that in the control group. Previous studies have shown that when 1-MCP is used to treat Asian pears (25) and salicylic acid is used to treat goji berries (5), the firmness of the treated fruit is higher than that of the untreated fruit during shelf life. This finding is consistent with our results. During the process of fruit ripening, the enzymes related to fruit softening are mainly polygalacturonase and cellulase, while 1-MCP and SA can delay the softening of winter jujube fruit, which may be related to the inhibition of the activities of these two enzymes, thereby delaying the decrease of fruit firmness (Figure 3C) (15).

### Response of SSC, TA content, and ethylene production of winter jujube to 1-MCP+SA treatment during shelf life

The SSC is an important index reflecting the postharvest maturity of winter jujube fruit. As shown in Figure 3D, the SSC of winter jujube fruit in the four groups increased first and then decreased. This may be due to the fact that there are large molecular substances, such as starch, in winter jujube fruit at the early stage of shelf life, and the degradation of these substances could increase the SSC of winter jujube fruit (26). At the later stage of shelf life, due to the nutrient consumption by respiration, the SSC decreased. Besides, our results showed that the SSC of fruit in the 1-MCP, SA, and 1-MCP+SA groups was 2.3, 3.1, and 5.4% higher than that in the control group on day 20, respectively (*P* < 0.05). This indicates that 1-MCP+SA treatment could effectively inhibit the decrease of SSC in winter jujube fruit. Organic acids in winter jujube fruit are important respiratory substrates to maintain its metabolism and have a vital effect on fruit flavor (4). As shown in Figure 3E, the TA content in winter jujube fruit in the four groups increased rapidly during the early stage of shelf life (day 0–4). This may be due to the fact that anaerobic respiration of fruit and metabolism of CO_2_ produced during respiration could produce a small amount of lactic acid and other substances during the shelf life, resulting in an increase in TA content (27). In the middle and later stages of shelf life, TA was further consumed as a substrate for respiration, resulting in a decrease in TA content (5). In this study, the 1-MCP+SA treatment inhibited the decrease in TA content more than the 1-MCP and SA treatments. This may be attributed to the joint action of ethylene inhibitor and natural plant growth inhibitor, which increases SSC and inhibits rapid decrease in TA content, thus delaying fruit senescence (4, 28).

Previous studies have shown that ethylene can regulate the signal molecules in plants. Besides, ethylene, as the only gas hormone among the five hormones in plants, can also extensively regulate seed dormancy and germination, root growth, shoot and leaf formation, flower development and fruit maturation, tissue aging, plant resistance, and the interaction with other plant hormones (15). As shown in Figure 3F, with the extension of shelf life, ethylene production in the control, 1-MCP, SA, and 1-MCP+SA groups showed an increasing trend in the beginning and then decreased, and reached the peak (1.89, 1.40, 1.34, and 1.18 μL kg^−1^ s^−1^, respectively) on day 16. At the same time, the ethylene production in the 1-MCP+SA treatment group (1.07 μL kg^−1^ s^−1^) was 20% lower than that in the control group (1.34 μL kg^−1^ s^−1^) on day 20. This indicates that 1-MCP+SA treatment could significantly reduce ethylene production during the shelf life of winter jujube fruit, delay fruit ripening and senescence, and maintain fruit quality.

### Response of MDA content, H_2_O_2_ content, and ${\text{O}}_{2}^{-\cdot }$ generation rate of winter jujube fruit to 1-MCP+SA treatment during shelf life

**Figure 4:**
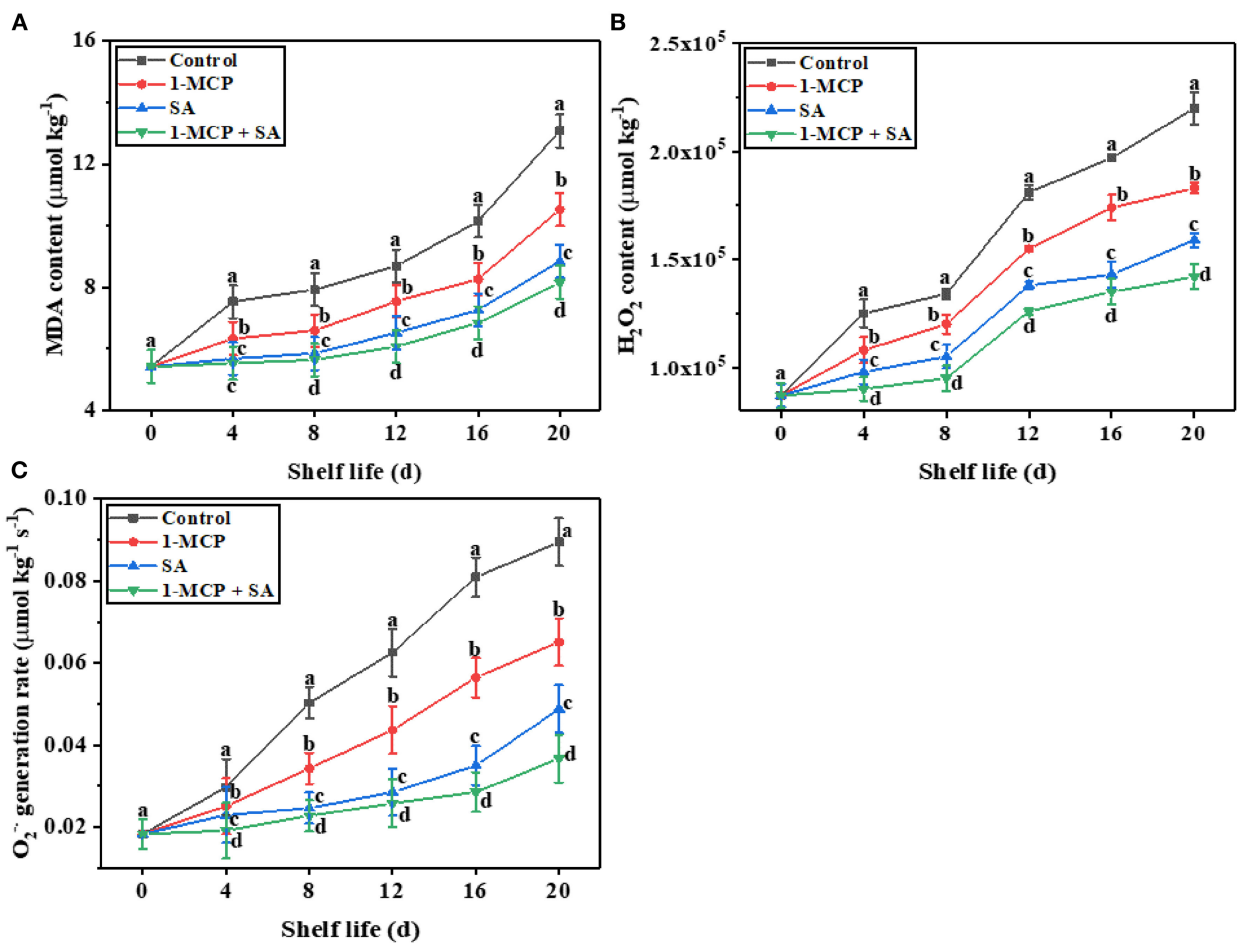
Effects of 1-methylcyclopropene (1-MCP), salicylic acid (SA), and 1-MCP and MDA content **(A)**, H_2_O_2_ content **(B)**, and O_2_^−·^ generate rate **(C)** of winter jujube fruit during shelf life at 4 ± 1°C for 20 d. MDA, malondialdehyde; H_2_O_2_, hydrogen peroxide; O_2_^−·^, superoxide anion. The data presented are the mean values of three replicates; vertical bars represent the standard errors of the mean values; values followed by different superscripts (a-d) are significantly different (*P* < 0.05) on the same sampling date.

Malondialdehyde is a cytotoxic metabolite produced by membrane lipid peroxidation during fruit storage, which can be used as an indicator of fruit senescence and membrane lipid peroxidation (29). In this study, with the extension of shelf life, the MDA content of fruit showed a rising trend in all the groups (Figure 4A). At the end of the shelf life, the MDA content in the control, 1-MCP, SA, and 1-MCP+SA groups were 13.08, 10.52, 8.84, and 8.15 umol·kg^−1^, respectively. The MDA content in the control group was 24.33, 47.96, and 60.24% higher than that observed in the 1-MCP, SA, and 1-MCP+SA groups, respectively (*P* < 0.05). Therefore, the degree of membrane lipid peroxidation in winter jujube fruit is different in different groups. It indicates that 1-MCP treatment and SA treatment could slow down the accumulation of MDA and inhibit membrane lipid peroxidation and fruit senescence of winter jujube. Hu et al. (11) showed that 1-MCP could effectively slow down the accumulation of MDA in cabbage, thus inhibiting lipid peroxidation. Siboza et al. (7) reported that SA treatment could improve the cold resistance and antioxidant activity of lemon fruit, and reduce the accumulation of MDA. These findings are similar to our experimental results. The reason for the reduced MDA content in the treated winter jujube fruit may be that SA and 1-MCP enhance the antioxidant capacity, thereby improving the cell membrane permeability and plasma membrane integrity in winter jujube fruit (29–31).

During oxidative metabolism, a large number of ROS, including H_2_O_2_ and ${\text{O}}_{2}^{-}$,^·^ are produced in fruit. ROS have a strong oxidative capacity and can destroy fruit biomembrane structure, leading to oxidative damage and fruit senescence (32). In this study, with the extension of shelf life, the H_2_O_2_ content in winter jujube fruit showed a rising trend. At the end of the shelf life, the content of H_2_O_2_ in the control group was 20.22, 38.36, and 54.93% higher than that in the 1-MCP, SA, and 1-MCP+SA groups (*P* < 0.05), respectively (Figure 4B). This indicates that SA and 1-MCP treatment could inhibit the production of H_2_O_2_ to varying degrees, and the inhibitory effect of 1-MCP+SA treatment was better than those of 1-MCP and SA treatments. Similar to our results, the H_2_O_2_ content of “Yali” pear (32) and goji berries (33) was also significantly reduced by 1-MCP and SA treatments. The reason for the reduced H_2_O_2_ content in the treated winter jujube fruit may be that 1-MCP and SA could enhance the activities of antioxidant enzymes such as APX, POD, SOD, and CAT, thus delaying fruit senescence and extending the shelf life of winter jujube fruit.

Chen et al. (34) pointed out that 1-MCP treatment significantly delayed the membrane lipid peroxidation of ‘Huanghua’ pear and inhibited the accumulation of ${\text{O}}_{2}^{-\cdot }$ in fruit after harvest. Zhang et al. (33) showed that SA treatment reduced the accumulation of ${\text{O}}_{2}^{-\cdot }$in goji berries. In this study, the effects of 1-MCP, SA, and 1-MCP+SA treatments on ${\text{O}}_{2}^{-\cdot }$ generation rate were similar to those on H_2_O_2_ (Figure 4C). At the end of the shelf life, the ${\text{O}}_{2}^{-\cdot }$ generation rate increased to 0.089, 0.065, and 0.049 μmol kg^−1^ s^−1^ in the control, 1-MCP, and SA groups, respectively, and the generation rate of ${\text{O}}_{2}^{-\cdot }$ in the 1-MCP+SA group was only half of that in the control group. This indicates that the 1-MCP+SA treatment could significantly inhibit the generation of ${\text{O}}_{2}^{-\cdot }$ in winter jujube fruit. The mechanism is similar to that of reduced H_2_O_2_. That is, the increased activities of antioxidant enzymes, such as SOD and CAT, in fruit reduce the ROS content (7).

### Impact of 1-MCP+SA treatment on SOD, POD, CAT, APX, GR, and PAL activity in winter jujube fruit during shelf life

**Figure 5:**
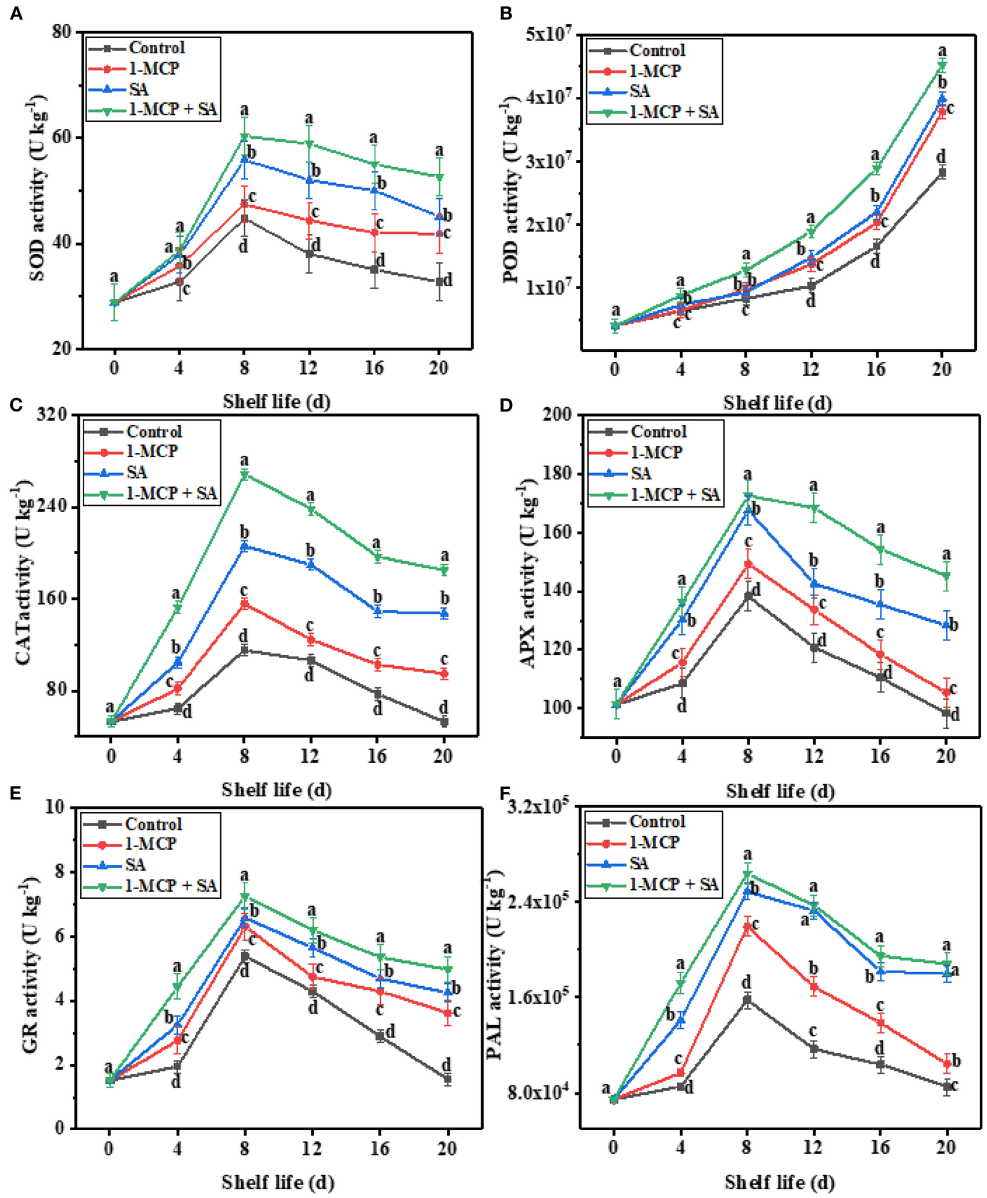
Effects of 1-methylcyclopropene (1-MCP), salicylic acid (SA), and 1-MCP and SA combined treatments on SOD **(A)**, POD **(B)**, CAT **(C)**, APX **(D)**, GR **(E)**, and PAL **(F)** activities of winter jujube during shelf life at 4 ± 1°C for 20 d. SOD, superoxide dismutase; POD, polyphenol oxidase; CAT, catalase; APX, ascorbic acid peroxidase; GR, glutathione reductase; PAL, phenylalanine ammonia-lyase. The data presented are the mean values of three replicates; vertical bars represent the standard errors of the mean values; values followed by different superscripts (a-d) are significantly different (*P* < 0.05) on the same sampling date.

Numerous studies have shown that during storage, the antioxidant defense system (e.g., SOD, POD, CAT, APX, GR, and PAL) in fruit can remove ROS produced during metabolism, reduce the oxidative damage caused by ROS to cells, and delay fruit senescence (33). SOD is an important ROS scavenging enzyme in plant enzymatic defense system, which can reduce the accumulation of ROS in fruit by catalyzing the transformation of ${\text{O}}_{2}^{-\cdot }$ into H_2_O_2_ in tissues (35). As shown in Figure 5A, the SOD activity in winter jujube fruit in the four groups increased gradually and reached a peak on day 8. Among them, the SOD activity of fruits in the 1-MCP+SA group was the highest. From day 8 to day 20, the SOD activity in all four groups decreased. At the end of shelf life (day 20), the SOD activity of fruit in the 1-MCP, SA, and 1-MCP+SA groups decreased to 41.81, 45.1, and 52.6 U·kg^−1^, respectively, which were significantly higher than that in the control group (32.75 U·kg^−1^) (*P* < 0.05). Xu et al. (31) showed that combined treatment of 1-MCP and SA effectively improved the SOD activity of banana fruit, inhibited the accumulation of ROS, slowed down fruit aging, and extended the shelf life. In addition, Lo'ay et al. (29) found that chitosan/polyvinyl alcohol-SA combined treatment could maintain high SOD activity in ‘Thompson Seedless’ grapes, which contributed to the rapid removal of ${\text{O}}_{2}^{-\cdot }$ accumulated in the fruit and inhibition of oxidative damage and fruit senescence. In our study, we found that the fruits in the 1-MCP+SA group had higher SOD activity and lower ROS (H_2_O_2_ and ${\text{O}}_{2}^{-\cdot }$) content (Figures 4B,C). This indicates that 1-MCP+SA combined treatment could improve the antioxidant capacity of winter jujube fruit.

The POD is a kind of oxidoreductase found in fruits. It can remove H_2_O_2_ produced in the metabolic process and maintain free radicals at a relatively low level by coordinating with SOD and other enzymes in fruit, so as to prevent the damage caused by the excess accumulation of free radicals and extend the shelf life of fruit (29). As shown in Figure 5B, the POD activity of winter jujube fruit in the four groups showed an increasing trend, while the activity in the 1-MCP+SA group was always maintained at a high level. At the end of shelf life, the POD activity of fruit in the 1-MCP, SA, and 1-MCP+SA groups reached the maximum, which were found to be 3.78 × 10^7^, 3.99 × 10^7^, and 4.52 × 10^7^ U·kg^−1^, respectively. However, the POD activity in the control group was only 2.83 × 10^7^ U·kg^−1^. Previous studies showed that 1-MCP and SA treatments delayed postharvest senescence of Xinjiang “Junzao” (2) and banana fruit (31), which was related to the regulation of POD activity. In line with these results, our study showed that 1-MCP+SA combined treatment increased POD activity and extended the shelf life of winter jujube fruit. This indicates that the regulation of antioxidant enzymes may be an important mechanism by which 1-MCP+SA combined treatment inhibits winter jujube fruit senescence.

Catalase can promote the decomposition of H_2_O_2_ into molecular oxygen and water, and protect fruit tissue cells from the toxicity of H_2_O_2_, thus prolonging the shelf life of fruit (18). In this study, the CAT activity in fruit in the four groups showed a similar trend. The CAT activity increased rapidly from day 0 to day 8 and then decreased slowly (Figure 5C). During the whole shelf life, the CAT activity in the control group was always lower than that in the other three groups, and the CAT activity in the 1-MCP+SA group was always at a high level. The CAT activity of fruit in the 1-MCP, SA, and 1-MCP+SA groups was 155.72, 206.05, and 268.57 U·kg^−1^, respectively, on day 8, which were 34.89, 78.49, and 132.65% higher than that in the control group, respectively (*P* < 0.05). At the end of shelf life, the CAT activity of fruit in the SA and 1-MCP+SA groups decreased to 147.49 and 184.96 U·kg^−1^, respectively, which were still higher than the highest CAT activity in the control group (115.44 U·kg^−1^). In conclusion, 1-MCP, SA, and 1-MCP+SA treatments could inhibit the decrease of CAT activity to varying degrees, and 1-MCP+SA treatment showed the best inhibitory effect, followed by SA treatment. CAT is a key enzyme to remove ROS, which protects plants from oxidative damage and delays fruit senescence (35). In previous studies, the 1-MCP treatment also led to higher antioxidant enzyme activities in yellow-fleshed kiwifruit (16), sweet cherry (17), and peach (36). Besides, 1-MCP in combination with SA also maintained higher activities of SOD, CAT, and APX in bananas (31). 1-MCP+SA treatment improved CAT activity, that is, the antioxidant capacity of fruits was improved. It may be that 1-MCP+SA treatment changed the redox state in cells, thus improving the activity of antioxidant enzymes (35). So, the combined treatment of 1-MCP and SA could be considered an efficient approach to maintaining high antioxidant enzyme activities in winter jujube fruit.

Ascorbate peroxidase is an important antioxidant enzyme that plays a synergistic role with CAT to convert H_2_O_2_ accumulated in fruit into H_2_O, to remove ROS. It can prevent fruit from damage caused by lipid peroxidation and extend its shelf life (18). Cheng et al. (2) found that the declining trend of APX activity in jujube fruit was inhibited by 1-MCP treatment. Other studies have shown that SA treatment could maintain higher activities of APX, SOD, CAT, and POD in melon (37) and sweet cherry (23). In our study, the APX activity in the four groups increased gradually and reached the maximum value on day 8 (Figure 5D), and the APX activity of fruit in the 1-MCP, SA, and 1-MCP+SA groups was 7.98, 21.21, and 24.44% higher than that in the control group (138.22 U·kg^−1^), respectively (*P* < 0.05). At the end of shelf life, the APX activity of fruit in the 1-MCP+SA group was 145.24 U·kg^−1^, which was significantly higher than the maximum value of APX activity in the control group (138.22 U·kg^−1^). This may be due to the interaction between antioxidant enzymes and the change in the content of antioxidant compounds, which can improve the antioxidant capacity and directly weaken the oxidative damage of fruit, and thus help to maintain the integrity of cell membrane structure, prolong the shelf life, and improve the preservation effect of fruit (18). Overall, the combined treatment of 1-MCP and SA could effectively maintain the antioxidant enzyme activity of winter jujube fruit.

Glutathione reductase is an important antioxidant enzyme that provides reductive power for scavenging ROS by reducing oxidized glutathione to reduced glutathione (30). It can protect from oxidative damage and prolong the shelf life of fruit (38). As shown in Figure 5E, the GR activity of winter jujube fruit in the 1-MCP, SA, and 1-MCP+SA groups increased slowly from day 0 to day 4, increased sharply from day 4 to day 8, and then began to decrease slowly until the end of shelf life. The GR activity of fruit in the 1-MCP, SA, and 1-MCP+SA groups was higher than that in the control group, and the 1-MCP+SA group showed the highest GR activity, followed by the SA group. On day 20, the GR activity in the 1-MCP (3.62 U·kg^−1^), SA (4.25 U·kg^−1^), and 1-MCP+SA (4.97 U·kg^−1^) groups was 132, 172, and 219% higher than that in the control group, respectively (*P* < 0.05). The above-mentioned results indicate that the 1-MCP+SA treatment could maintain a higher GR activity. This may be due to the synergistic action of SA and regulation of the intracellular redox state by 1-MCP, which leads to a significant change in the enzyme activity of GR (35). Previous studies have shown that SA treatment could increase the activity of antioxidant enzymes in cucumber (30) and lemon (7), thereby enhancing the cold tolerance of fruit during storage. Also, 1-MCP treatment could increase the GR activity in apple (39) and ‘Yujinxiang’ melon (38). These findings further demonstrate that the effects of SA and 1-MCP are consistent in improving the antioxidant activity of fruit.

Phenylalanine ammonia-lyase is an important enzyme that is involved in the phenylpropane pathway in plants. It is closely related to disease resistance, stress resistance, and the synthesis of secondary metabolites, such as total phenols, anthocyanins, and flavonoids (33). As shown in Figure 5F, the PAL activity of winter jujube fruit in the four groups showed an increasing trend and reached the peak on day 8: [1.58 × 10^5^ (control group), 2.19 × 10^5^ (1-MCP), 2.48 × 10^5^ (SA), and 2.63 × 10^5^ U·kg^−1^ (1-MCP+SA)]. At the end of the shelf life, the PAL activity of fruit in the control group and the 1-MCP group decreased sharply, quicker than that observed in the SA and 1-MCP+SA groups. Therefore, the preservation effect of SA treatment was better than that of 1-MCP treatment. Meanwhile, the PAL activity of fruit in the 1-MCP, SA, and 1-MCP+SA groups were 22.41, 110.99, and 120.5% higher than that in the control group, respectively (*P* < 0.05). SA and 1-MCP+SA treatments could significantly delay the decrease of PAL activity, while 1-MCP treatment had no significant effect (*P* > 0.05). The reason for the increase in PAL activity in winter jujube may be that the fruit treated exogenous 1-MCP and SA as a kind of stress, and thus enhanced their resistance response. A previous study has shown that SA treatment can maintain high PAL activity in apple leaves, which is helpful for plants to better resist stress conditions (40). Besides, 1-MCP treatment could improve the activity of PAL in postharvest blueberry fruit (41). The results of this experiment also confirm this view. In addition, in this study, it was observed that the PAL activity of fruit in the 1-MCP+SA group was not significantly different from that in the SA group (*P* >0.05) at the end of shelf life. The reason may be that the concentration of 1-MCP selected in the experiment was too high, which led to a decline in the PAL activity and stress resistance of the fruit (42). De Reuck et al. (42) found that a higher concentration of 1-MCP could reduce the membrane integrity of litchi fruit and accelerate fruit browning. Therefore, the concentration of 1-MCP and SA in the combined treatment can be further optimized. But, the 1-MCP and SA concentrations used in the 1-MCP+SA treatment in this study can effectively maintain the quality of winter jujube fruit.

### Impact of 1-MCP+SA treatment on non-enzymatic antioxidants in winter jujube fruit during shelf life

**Figure 6:**
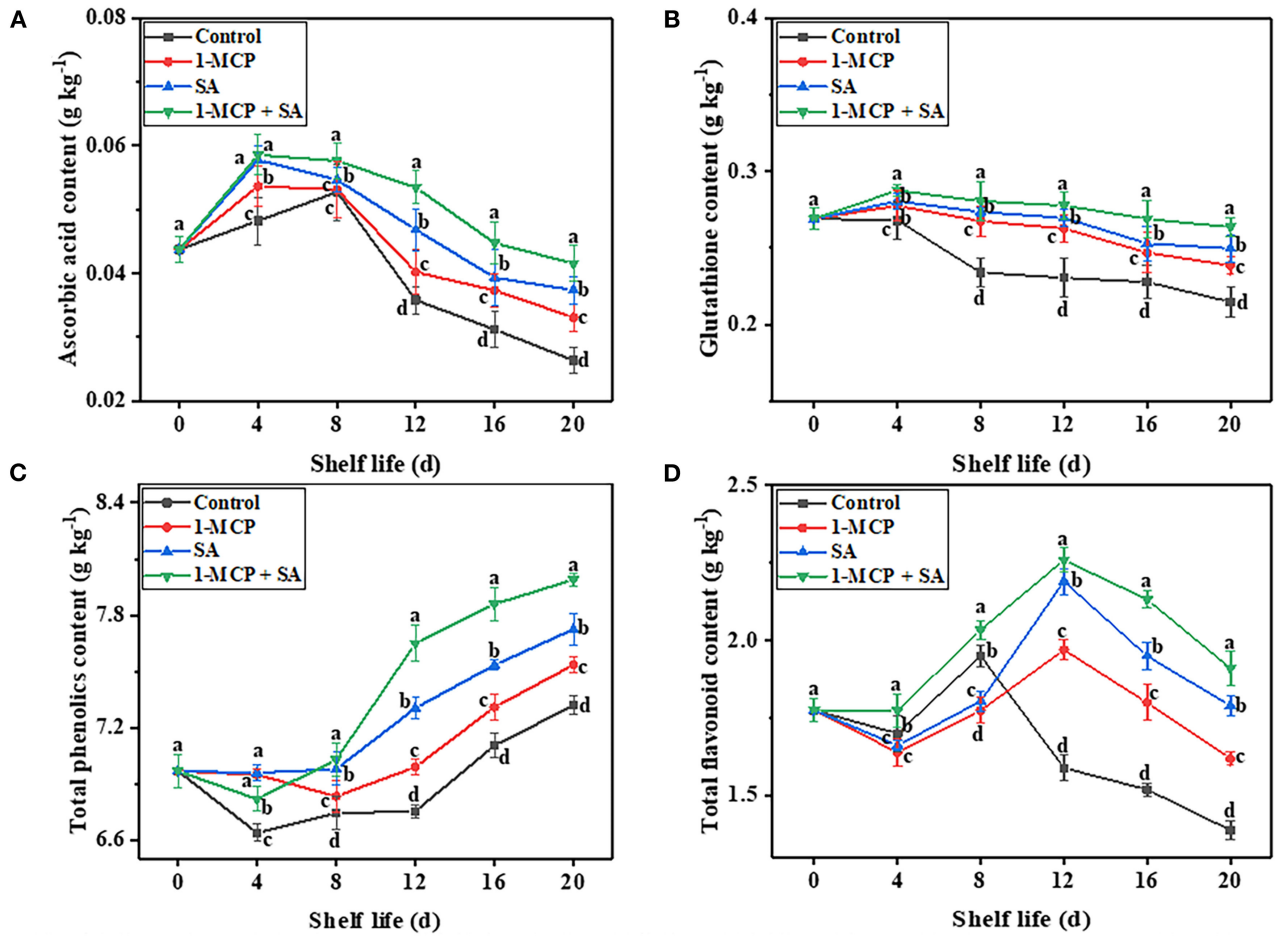
Effects of 1-methylcyclopropene (1-MCP), salicylic acid (SA), and 1-MCP and SA combined treatments on ascorbic acid content **(A)**, glutathione content **(B)**, total phenolics content **(C)**, and total flavonoid content **(D)** of winter jujube fruit during shelf life at 4 ± 1 °C for 20 d. The data presented are the mean values of three replicates; vertical bars represent the standard errors of the mean values; values followed by different superscripts (a-d) are significantly different (*P* < 0.05) on the same sampling date.

Previous studies have found that non-enzymatic antioxidants, such as ascorbic acid, glutathione, flavonoids, and phenols, play an important role in the resistance of plants to excessive ROS accumulation by coordinating with antioxidant enzymes to maintain the balance of the antioxidant system (3). In this study, 1-MCP+SA treatment maintained the balance of ROS metabolism in winter jujube fruit. For example, the high levels of ascorbic acid, glutathione, total flavonoids, and total phenolics in the 1-MCP+SA group (Figures 6A–D) resulted in the ability of the fruit to scavenge the continuously accumulated H_2_O_2_ and ${\text{O}}_{2}^{-\cdot }$ (Figures 4B,C), leading to a low accumulation of H_2_O_2_ content and ${\text{O}}_{2}^{-\cdot }$ generation rate at the end of storage. Therefore, the higher content of non-enzyme antioxidants at the later shelf life may also result in a better defense capability of winter jujube fruit. Previous studies on melon (38), goji berry (33), cabbage (11), and apricot (9) have also found that there is an important association between the non-enzymatic antioxidants and ROS metabolism in fruits treated with 1-MCP and SA. These results indicate that 1-MCP and SA are helpful to maintain the balance of ROS metabolism in winter jujube fruit during shelf life.

### Correlation analysis of fruit quality indexes and reactive oxygen metabolism in winter jujube fruit under 1-MCP+SA treatment

**Figure 7:**
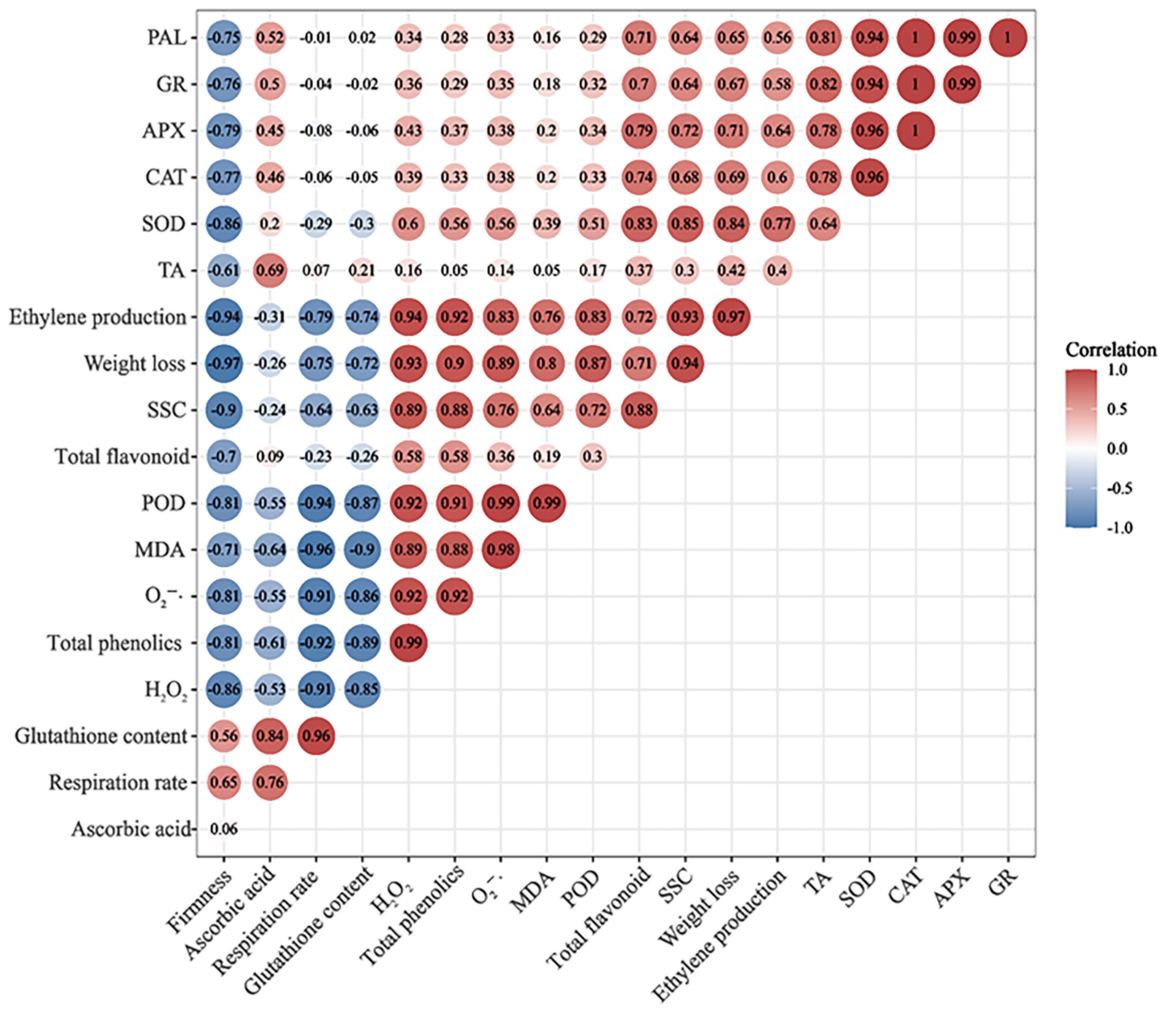
Correlation analysis of fruit quality indexes and reactive oxygen metabolism in 1-MCP+SA treated winter jujube fruit. Dark color represents a strong correlation, light color represents a weak correlation, red color represents a positive correlation, and blue color represents a negative correlation.

**Figure 8:**
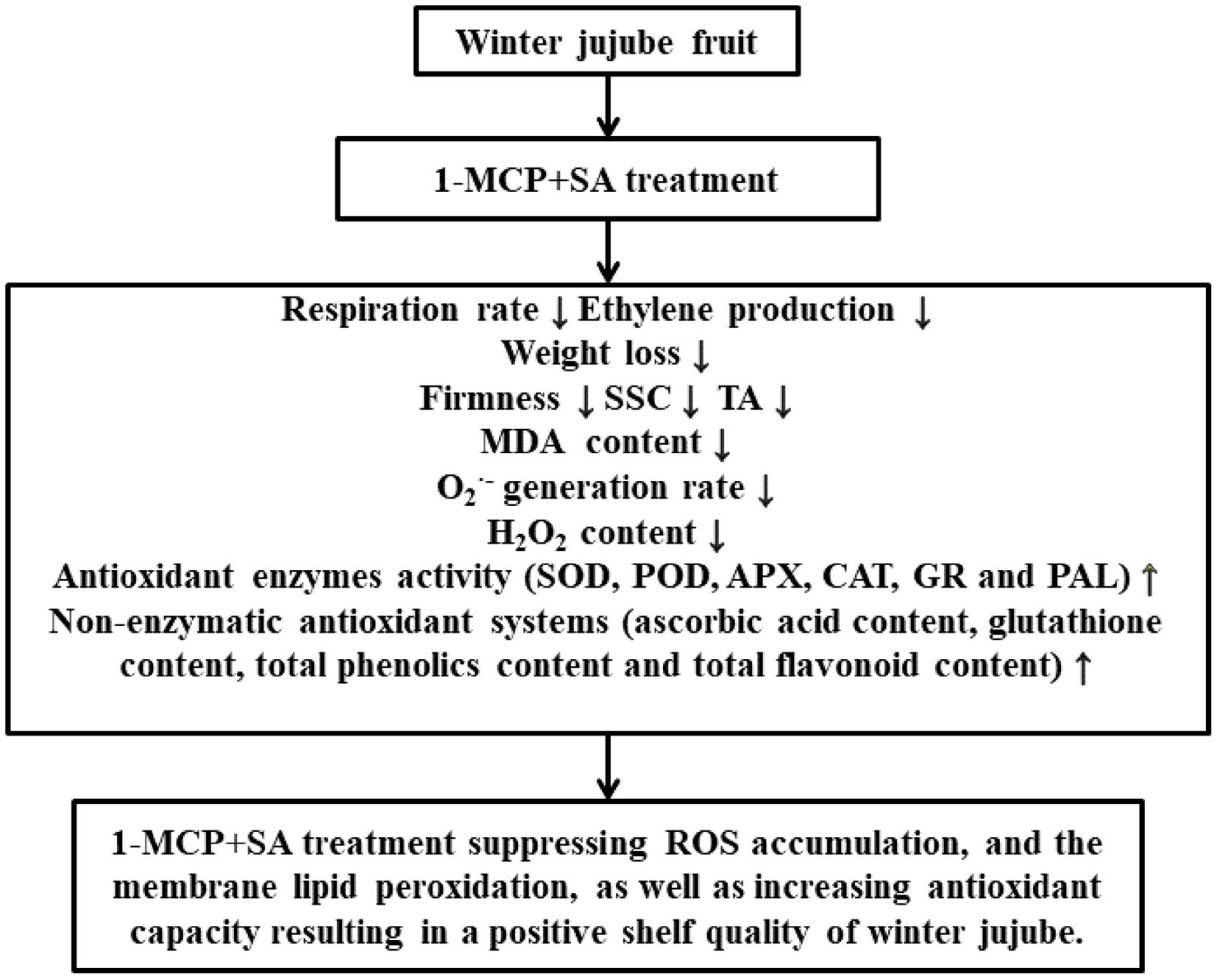
Mechanism of 1-MCP+SA treatment in improving quality indexes and antioxidant defense system of winter jujube fruit.

The ROS mainly include ${\text{O}}_{2}^{-\cdot }$, H_2_O_2_, and singlet oxygen (43). In response to ROS accumulation, plants have developed antioxidant defense systems, which include the production of non-enzymatic antioxidants (e.g., ascorbic acid, glutathione, anthocyanins, flavonoids, and phenolic compounds) and antioxidant enzymes (POD, SOD, CAT, APX, PAL, and GR) (3). Under normal conditions, there is a balance between the production and scavenging of ROS (18). As the fruit ripens, the ROS scavenging ability of these defense systems decreases, leading to increased ROS content, cell membrane damage, and fruit aging and softening (9). In this study, according to the Pearson correlation coefficients (Figure 7), both H_2_O_2_ content and ${\text{O}}_{2}^{-\cdot }$ generation rate were positively correlated with MDA content, total phenolics, total flavonoid content, and the activities of PAL, GR, APX, CAT, SOD, and POD. This may be due to the fact that with the gradual aging of winter jujube fruit, its ability to scavenge ROS decreases, leading to the increased H_2_O_2_ content, ${\text{O}}_{2}^{-\cdot }$ generation rate, and MDA content. However, the increase in H_2_O_2_ content and ${\text{O}}_{2}^{-\cdot }$ generation rate could cause oxidative stress in winter jujube. Therefore, the activity of antioxidant enzymes (such as PAL, GR, APX, CAT, SOD, and POD) and the content of non-enzymatic antioxidants are further enhanced in the fruit, thereby inhibiting excessive accumulation of H_2_O_2_ and ${\text{O}}_{2}^{-\cdot }$(Figure 8). During this process, 1-MCP+SA treatment could improve the antioxidant defense system of winter jujube fruit and delay fruit senescence.

Fruit firmness was negatively correlated with SSC (r = −0.90, *P* < 0.05), TA (r = −0.61, *P* < 0.05), and weight loss (r = −0.97, *P* < 0.01), whereas respiration rate was positively correlated with firmness (r = 0.65, *P* < 0.05) (Figure 7). These results indicate that SSC and TA are consumed in the process of ripening and softening of winter jujube fruit for transpiration and respiration, resulting in an increase in weight loss (44).

## Conclusion

The combined treatment of 1-MCP and SA is an effective method to prolong the shelf life of winter jujube fruit while maintaining fruit quality. 1-MCP+SA treatment could suppress weight loss, respiration, and ${\text{O}}_{2}^{-\cdot }$ generation, decrease MDA and H_2_O_2_ content, and maintain the firmness, Δ*E*, SSC, and TA. It can also maintain the activities of antioxidant defense enzymes, such as SOD, POD, CAT, APX, GR, and PAL, and the content of non-enzymatic antioxidant substances, such as ascorbic acid, glutathione, phenolics, and flavonoid, in fruits at a high level. Therefore, the combined treatment of 1-MCP and SA can be widely applied in the preservation of winter jujube fruit to extend the shelf life.

## Data Availability Statement

The original contributions presented in the study are included in the article/supplementary material, further inquiries can be directed to the corresponding authors.

## Author contributions

WZ: writing of the original draft, methodology, and software. JK: data curation, methodology, formal analysis, and investigation. WY and HG: data curation, formal analysis, and investigation. MG: conceptualization, supervision, reviewing and editing, validation, and funding acquisition. GC: conceptualization, supervision, and funding acquisition. All authors contributed to the article and approved the submitted version.

## Funding

This work was financially supported by the High-Level Talents Research Start Project of Shihezi University (RCZK201940) and the Innovation Project for the South-Forward Development of Xinjiang Production and Construction Corps (2018DB002).

## Conflict of interest

The authors declare that the research was conducted in the absence of any commercial or financial relationships that could be construed as a potential conflict of interest.

## Publisher's note

All claims expressed in this article are solely those of the authors and do not necessarily represent those of their affiliated organizations, or those of the publisher, the editors and the reviewers. Any product that may be evaluated in this article, or claim that may be made by its manufacturer, is not guaranteed or endorsed by the publisher.
